# Coaxial Electrospraying for Black Seed Oil Nanoencapsulation: Improved Thymoquinone Stability and Bioactivity

**DOI:** 10.1002/fsn3.70622

**Published:** 2025-09-15

**Authors:** Elif Atay, Aylin Altan, Derya Yetkin, Furkan Ayaz

**Affiliations:** ^1^ Department of Food Engineering Mersin University Mersin Türkiye; ^2^ Department of Histology and Embryology Mersin University Mersin Türkiye; ^3^ Department of Molecular Biology and Genetics, Faculty of Engineering and Natural Sciences Biruni University Istanbul Türkiye

**Keywords:** bioaccessibility, bioavailability, black seed oil, coaxial electrospraying, inflammation, nanoparticles, thymoquinone

## Abstract

Thymoquinone is the primary bioactive compound in black seed oil (BSO), but it has limited stability under harsh conditions. Nanoencapsulation using zein via coaxial electrospraying is an effective approach to improve its stability and bioactivity during gastrointestinal transit. Therefore, this study aimed to develop BSO‐loaded nanoparticles using coaxial electrospraying, evaluate their stability at different temperatures (4°C, 25°C and 60°C), and investigate their in vitro bioaccessibility, intestinal permeability using Caco‐2 cell models, and immunomodulatory potential. Nanoparticles with high encapsulation efficiency (74.38% ± 2.6%) and loading capacity (12.99% ± 3.6%) retained thymoquinone content at 91.3%, 74.7%, and 52.0% after 55 days of storage at 4°C, 25°C, and 60°C, respectively. The bioaccessibility of thymoquinone in nanoparticles increased from 21.7% to 33.1% when orange juice was fortified with nanoparticles. The apparent permeability of thymoquinone in nonencapsulated oil, nanoparticles, and orange juice fortified with nonencapsulated oil and nanoparticles was 2.10 × 10^−7^, 5.76 × 10^−7^, 4.39 × 10^−7^, and 7.38 × 10^−7^ cm/s, respectively. When the intracellular signaling pathways were analyzed for its intracellular mechanism of action, thymoquinone nanoparticles affected the activation levels of the p38 MAPK and PI3K proteins and stimulated macrophages to generate substantial levels of pro‐inflammatory cytokines, including TNFα, IL6, GMCSF, and IL12p40. BSO‐loaded nanoparticles can be incorporated into functional food formulations to deliver health benefits.

## Graphical abstract

The shell enhanced the stability of thymoquinone during storage at high temperature. The bioaccessibility of thymoquinone‐loaded nanoparticles increased with encapsulation. The transport of thymoquinone across Caco‐2 monolayers was improved by fortification.

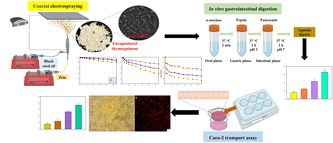

## 1 Introduction

The seeds of *Nigella sativa* L., often referred to as black seed, have a long history of use as a traditional remedy for improving health and addressing a wide range of diseases. Black seed oil (BSO), extracted from black seeds, contains bioactive compounds that are linked to properties such as antimicrobial, antidiabetic, antiulcer, and anticancer effects (Edris et al. 2016). Thymoquinone is one of the lipophilic bioactive compounds in BSO that has received particular attention for its beneficial role in various human metabolic processes. However, its strong hydrophobic nature makes it challenging to incorporate into many products and limits its absorption in the gastrointestinal tract (GIT). In recent years, the incorporation of bioactive compounds such as thymoquinone into functional foods has been of major interest due to their potential health‐promoting properties such as anticancer, antioxidant, analgesic, antimicrobial, anti‐inflammatory, and gastro‐protective influences (İnce et al. 2020). Despite the well‐known beneficial effects of BSO and its thymoquinone compound, its poor aqueous solubility, low bioavailability, instability during storage or digestion conditions, poor chemical stability, and sensitivity to environmental factors such as oxygen, light, temperature, moisture, ions, and acidic and basic conditions make it difficult to use in commercial food products (Dawaba and Dawaba 2019). To overcome these limitations, various encapsulation techniques have been explored to encapsulate black seed oil using different carriers such as yeast cell, gum arabic‐inulin, and hydrogel beads (Karaman 2020; Santiworakun et al. 2022; Wong et al. 2021). Among these techniques, the most common one is spray drying, but there are still limitations regarding its application in the encapsulation of volatile or heat‐sensitive bioactive compounds (Souza Simões et al. 2017). Therefore, there is a need for another encapsulation technique that can protect volatile bioactive compounds during storage, resist harsh gastric conditions, and control the release of these compounds during gastrointestinal (GI) passage. Coaxial electrospraying has emerged as a highly effective method for producing core‐shell nanoparticles, which provide a protective barrier around sensitive compounds. Therefore, nanoparticles obtained through nanoencapsulation of BSO offer a promising strategy for preserving thymoquinone and enabling its use in food products.

Nanoencapsulation, an advanced encapsulation technique, is one of the most effective approaches for stabilizing sensitive bioactive compounds, controlling their release in the gastrointestinal tract, and thus enhancing the bioavailability. Studies suggest that nanoencapsulated bioactive compounds are more easily absorbed by cells, thereby enhancing the delivery efficiency, nutritional value, and health benefits of targeted bioactive compounds (Jayan et al. 2019; Gonçalves et al. 2023; Finos et al. 2024). The electrospraying process, also known as electrohydrodynamic atomization, can be used as an effective nanoencapsulation method and offers several advantages for improving the stability of bioactive compounds, enabling sustained release, and enhancing bioavailability (Shishir et al. 2018; Mahalakshmi et al. 2020). This process has gained increased interest in the nanoencapsulation of bioactive compounds. It is suitable for the nanoencapsulation of volatile and sensitive compounds due to the nonthermal process. Coaxial electrospraying involves the use of two capillaries or needles coaxially placed together to develop particles with a core‐shell structure (Shishir et al. 2018). The core solution containing the bioactive compound is pumped through the inner needle, whereas the shell solution, typically a polymer material, is pumped through the outer needle. At the tip of the nozzle, the two solutions meet concentrically and are atomized under the influence of an electric field. As a result, droplets with a core‐shell structure are formed. The core‐shell particles produced through this technique exhibit enhanced stability and controlled release properties, making it a valuable method for delivering bioactive compounds. This technique is also a cost‐effective and simple technology for producing core‐shell shaped nanoparticles in a single step. Furthermore, zein‐based nanoparticles could protect the loaded compounds and improve stability and bioactivity during digestion (Luo et al. 2023). Therefore, zein‐based nanoparticles serve as promising shell materials for protection in the gastrointestinal tract.

The Caco‐2 cell model is commonly used in conjunction with in vitro simulated digestion to evaluate the bioaccessibility and potential absorption of nutrients and bioactive compounds. Caco‐2 cells are derived from human colon carcinoma and exhibit similar characteristics to human intestinal enterocytes when they differentiate after 14–21 days after being seeded, making them a valuable tool for simulating the functional and morphological properties of the human intestinal barrier (Santos et al. 2019). A murine macrophage cell line that produces levels of the pro‐inflammatory cytokines TNFα, IL6, GMCSF, and IL12p40 can also be used to determine the immunomodulatory potential of bioactive compounds (Ayaz et al. 2021; Yüzer et al. 2019).

Considering the nutritional and metabolic significance of BSO, it is essential to further explore the effect of nanoencapsulation on its chemical and biological characteristics. Furthermore, investigating thymoquinone behavior in nanoencapsulated BSO is essential for exploring new opportunities in functional food development. This research introduces the innovative use of electrospraying for the nanoencapsulation of BSO, resulting in improved thymoquinone stability and bioactivity. The effects of electrospraying conditions and solution properties on the structure of core‐shell shaped nanoparticles and the stability of BSO during storage have been investigated by our research group (Atay and Altan 2021). However, cellular uptake of thymoquinone from BSO nanoparticles using an in vitro digestion model combined with Caco‐2 cells, as well as the immunomodulatory effect, were not assessed. Thymoquinone has previously been nanoencapsulated using chitosan, nanostructured lipid carriers, and chitosan‐modified polycaprolactone via ionic gelation, high‐pressure homogenization, solvent evaporation, and nanoprecipitation, primarily for pharmaceutical applications (Alam et al. 2012; İnce et al. 2020; Ansar et al. 2020; Rahat et al. 2021). However, no study to date has investigated the bioaccessibility and delivery efficacy of thymoquinone from BSO in the presence of a food matrix. Therefore, the present study aims to explore bioaccessibility, transepithelial transport across Caco‐2 monolayers, and the immunomodulatory potential of thymoquinone encapsulated in core‐shell zein nanoparticles, as well as its fate when incorporated into orange juice fortified with BSO‐loaded nanoparticles.

## 2 Materials and Methods

### 2.1 Materials, Chemicals and Reagents

BSO was provided by local producer (Çakıroğlu Oil LLC Co) in Mersin, Türkiye. α‐amylase (1000 U mg^−1^), pepsin (250 U mg^−1^), pancreatin (4 U mg^−1^), Tween 80 (P1754), bile extract (100 mmol g^−1^), zein protein (grade Z3625) and thymoquinone (274666) were purchased from Sigma‐Aldrich (St. Louis, MO, USA). The human cell line Caco‐2 (passage #23) was supplied by the Republic of Türkiye Ministry of Agriculture and Forestry (Ankara, Türkiye). High glucose Dulbecco's Modified Eagle's Medium (DMEM, 41966‐052), penicillin/streptomycin (15140), GlutaMAX (35050‐038), phosphate buffered saline (PBS), 0.25% trypsin–EDTA solution, nonessential amino acid mixture (MEM NEAA, 11140‐035), fetal bovine serum (FBS) and Hank's balanced salt solution (HBSS) were all obtained from Gibco (Waltham, Massachusetts, USA). Oranges were purchased from a local market in Mersin, Türkiye.

### 2.2 Experimental Design

An experimental design using the *Box–Behnken* method was employed to produce BSA‐loaded zein particles (Design‐Expert, v7.0, Stat‐Ease, Minneapolis, MN, USA) (Atay and Altan 2021) (Table S1). The independent variables selected for the study were shell solution concentration (14%, 17% and 20% w/v), flow rate of core solution (0.40, 0.55 and 0.70 mL h^−1^), distance between collector and needle (14.0, 15.5 and 17.0 cm), and applied voltage (15.0, 16.5 and 18.0 kV). A total of 29 runs were conducted to evaluate the effects of the independent factors on dependent variables such as loading capacity and encapsulation efficiency of thymoquinone.

### 2.3 Preparation of Zein Nanoparticles Containing BSO

The shell solution was prepared by dissolving zein in a solvent mixture consisting of glacial acetic acid and ethanol (1:1, v/v), whereas the core solution was made by dissolving 10% (w/v) zein in 80% (v/v) ethanol, followed by the addition of 10% (w/v) whey protein isolate. The core solution was then blended with BSO (1:1) and Tween 80 (5%, v/v). The core solution was homogenized at 700 rpm for 3 min using a homogenizer (HG‐15D, WiseTis, Korea), maintained at a temperature of 25°C ± 5°C.

### 2.4 Coaxial Electrospraying Process

Core‐shell nanoparticles containing BSO were produced using a coaxial electrospraying system (NE 100, Inovenso, Türkiye) according to the experimental design (Table S1). Nanoparticles were produced using electrospraying with two concentric stainless steel nozzles, having inner diameters of 0.8 and 1.2 mm. The distance between the needle and collector, the core solution flow rate, and the applied voltage were varied based on the experimental design. The flow rate of the shell solution and core solution concentration were maintained at 1 mL h^−1^ and 10% (w/v), respectively.

### 2.5 Encapsulation Efficiency and Loading Capacity

For the assessment of encapsulation efficiency and loading capacity of thymoquinone, BSO was extracted with hexane (5 mL) from nanoparticles by dissolving them in 3 mL of 80% ethanol (Atay and Altan 2021). The thymoquinone content in extracted oil and surface oil, present on the surface of nanoparticles, was determined using a gas chromatography (7890A, Agilent Technologies, USA) and flame ionization detector equipped with capillary column (19091N‐1331 HP‐INNOWAX; 30 m × 0.25 mm × 0.25 mm). The oven temperature was programmed as follows: heated from 70°C to 260°C at a rate of 20°C min^−1^, then held at 260°C for 10 min. The inlet and detector temperatures were set at 270°C and 330°C, respectively. The carrier gas was helium (1.7 mL min^−1^). The amount of thymoquinone was determined from a calibration curve (*y* = 1.5429*x* + 2.1245, *R* ^2^ = 0.9998), which was constructed using varying concentrations of thymoquinone dissolved in hexane. Equations (1) and (2) were used to calculate encapsulation efficiency (EE) and loading capacity (LC):

(1) $$\mathrm{EE}\;\left(\%\right)=\frac{T_{A}-T_{B}}{T_{A}}\times 100$$

(2) $$\mathrm{LC}\;\left(\%\right)=\frac{T_{A}-T_{B}}{P}\times 100$$

where *T*_A_ is the amount of total thymoquinone entrapped in the nanoparticles (mg), *T*_B_ indicates the quantity of thymoquinone located on the nanoparticle surface (mg) and *P* refers to the total mass of the nanoparticles (mg).

### 2.6 Thymoquinone Stability in BSO‐Loaded Nanoparticles During Storage

Nanoparticles with the highest (B1) and the lowest (W1) encapsulation efficiency of thymoquinone were selected from 29 runs to determine storage stability of thymoquinone under different storage conditions. BSO‐loaded zein nanoparticles and free BSO were placed in sealed vials and stored for 55 days under three storage conditions: 4°C with 39.8% ± 1.5% relative humidity (RH) representing refrigerated storage, 25°C with 26.7% ± 1.2% RH representing ambient conditions, and 60°C with 10.6% ± 2.1% RH representing accelerated storage testing. The amount of thymoquinone retained in the nanoparticles and oil was determined by gas chromatography during the 55 days of storage as described in Section 2.3. The retention of thymoquinone during storage was defined as the thymoquinone content in the nanoparticles to the initial thymoquinone content.

### 2.7 Fortification of Orange Juice With BSO‐Loaded Nanoparticles

Orange juice is liquid food commonly used as vehicles for oil fortification (Stefani et al. 2019). Therefore, orange juice was chosen as a food matrix to assess the behavior of thymoquinone within the nanoparticles. The sample with the highest encapsulation efficiency was used for the fortification of cloudy orange juice with thymoquinone‐loaded zein nanoparticles. The suggested intake doses of thymoquinone are 5–12.5 mg kg^−1^ for anticancer, anti‐inflammatory, and antioxidant effects (Abedi et al. 2016). Based on this recommended dose, 5 mL of cloudy orange juice was fortified with 0.32 mL BSO and 100 mg of BSO‐loaded nanoparticles, and homogenized until complete dissolution at 700 rpm for 3 min (HG‐15D, WiseTis, Korea).

### 2.8 Bioaccessibility of Thymoquinone

Thymoquinone bioaccessibility assessed in vitro within the gastrointestinal tract in core‐shell structured nanoparticles and nonencapsulated oil was determined using the INFOGEST method standardized by Brodkorb et al. (2019). During in vitro digestion, compound 1: BSO, black seed oil (0.32 mL oil in 5 mL); compound 2: BSO‐oj, orange juice enriched with nonencapsulated BSO (0.32 mL oil in 5 mL); compound 3: NPs, nanoparticles (100 mg nanoparticles in 5 mL); compound 4: NPS‐oj, orange juice enriched with nanoparticles (100 mg nanoparticles in 5 mL) were used to determine the in vitro GI bioaccessibility of thymoquinone. In vitro GI digestion steps were carried out according to our previous study (Atay and Altan 2021). Samples were subjected to simulated digestion through the oral, gastric, and intestinal phases. The simulated salivary fluid (SSF), gastric fluid (SGF) and intestinal fluid (SIF) were prepared using the INFOGEST protocol. The oral phase digestion started by adding 4 mL of SSF, 0.5 mL of α‐amylase solution, 0.475 mL of water, and 25 μL of 0.3 M CaCl_2_ to the sample and the incubation of the mixture for 2 min at 37°C and 100 rpm (WiseCube, WIS‐20, Germany). In the gastric phase, the bolus sample was mixed with 4 mL of the SGF, 0.25 mL of pepsin solution, 0.398 mL of water, and 2.5 μL of 0.3 M CaCl_2_ solution. After pH adjustment with 1 M HCl (pH: 3.0), the mixture was shaken continuously at 37°C for 2 h in an incubator. Afterwards, the intestinal phase digestion was performed by mixing the gastric chyme with 4.25 mL of the SIF, 2.5 mL of pancreatin solution, 1.25 mL of bile solution, 1.78 mL of water, and 20 μL of 0.3 M CaCl_2_. The pH of the solution was brought to 7.0 using 1 M NaOH, followed by incubation at 37°C for 3 h under continuous agitation at 100 rpm. After in vitro digestion, samples were freeze‐dried and dissolved in hexane for GC analysis. The amount of thymoquinone in digested samples was measured as described in Section 2.5. The in vitro bioaccessibility of thymoquinone was calculated using Equation (3):

(3) $$\text{Bioaccesibility}\;\left(\%\right)=\frac{C_{D}}{C_{T}}\times 100$$

where *C*_D_ and *C*_T_ represent the amount of thymoquinone determined after and before in vitro digestion (mg), respectively.

### 2.9 Cell Culture

Caco‐2 cells were preserved in DMEM containing 1% of nonessential amino acids solution, 10% fetal bovine serum, and 1% penicillin/streptomycin, and 1% GlutaMAX solution in 75 cm^2^ plastic dishes. Viable and nonviable cells were counted to calculate cell viability using the trypan blue dye exclusion method (Saleh et al. 2021). An aliquot of cell suspension being tested for viability was centrifuged (100 *g*, 5 min) and the cell pellet was resuspended in PBS. Cell suspension (1 mL) and trypan blue (0.4%) were mixed in the same volume to dilute the cells. Then, the trypan blue/cell mixture was incubated at room temperature for 3 min. Cell viability was determined using a hemocytometer on a drop of the mixture. Cell viability was calculated using Equation (4):

(4) $$\text{Cell viability}\;\left(\%\right)=\frac{\text{number of live cells}}{\text{total number of cells}}\times 100$$

Caco‐2 cells were seeded (2.5 × 10^5^ cells cm^−2^) in 6‐Transwell polyester insert plates (Greiner Bio‐One GmbH, Kremsmünster, Austria) with a growth area of 4.52 cm^2^ and pore size of 0.4 μm in a humidified atmosphere of air/CO_2_ (95:5) at 37°C. The DMEM was changed every 2 days, and the transport assays were performed 21 days post‐seeding to make them differentiate into mature enterocytes. Parameters such as inverted microscope image, dye leakage of methylene blue, and transepithelial electrical resistance (TEER) were examined to confirm cell monolayer integrity (Taboada‐Lopez et al. 2021; Boim et al. 2020). The evaluation via inverse microscopy (Axio Vert A1, Carl Zeiss, Germany) at 40× magnification was carried out to monitor the cell integrity and differentiation of monolayers (Boim et al. 2020). To determine the monolayer integrity of cells using the methylene blue test, methylene blue (5 mg L^−1^) was added from the apical chamber and left for 120 min (Taboada‐Lopez et al. 2021). The absorbance of dye leakage was measured with a spectrophotometer at 664 nm, and dye leakage was determined using a calibration curve of methylene blue (*y* = 0.2086*x* + 0.0691, *R* ^2^ = 0.9973). Less than 2% methylene blue dye leakage was considered proof of the integrity of the cell monolayer. To ensure monolayer integrity, only wells with less than 25% change of TEER value were used in the experiments. TEER values of Caco‐2 cells were measured using the voltohmmeter (BM812X, Breymen, Taiwan) with a silver/silver chloride electrode suitable for cell tissue. TEER values of cells were determined by calculating the difference between blank HBSS fluid and Caco‐2 cells with HBSS fluid (Equation (5)):

(5) $$\text{TEER}=\left(R_{\text{total}}-R_{\text{blank}}\right)\times A$$

where *R*_total_ is the measured resistance (Ω) across the cell layer, *R*_blank_ is the resistance of blank Transwell (without cells) (200 Ω), A is the area of Transwell filter (4.52 cm^2^) (Srinivasan et al. 2015).

#### 2.9.1 Transport Assay to Estimate Intestinal Absorption of Thymoquinone

The Caco‐2 cells used in the transport assay were seeded and differentiated in 6‐Transwell polyester insert plates due to the protocol given above. For the transport assay, DMEM used as an incubation medium was aspirated and HBSS solution (2.5 mL) containing nonencapsulated oil, orange juice fortified with nonencapsulated oil, nanoparticles, and orange juice fortified with nanoparticles was added to the apical side, while the blank HBSS solution (2 mL) was added to the basolateral side. Samples (100 μL) were taken from the basolateral side after 30, 60, 120, and 180 min and replaced with the same volume of HBSS. At the beginning and end of the experiment, TEER values were also determined according to the protocol given above. The amount of thymoquinone in samples was measured as described in Section 2.3. The apical to basolateral apparent permeability (*P*_app_, cm s^−1^) was calculated according to Equation (6):

(6) $$P_{\mathrm{app}}=\frac{dQ}{dt}\times \frac{V}{AC_{0}}$$

where d*Q*/d*t*, *A*, *V*, and *C*_0_ represent the slope of cumulative concentration (mg thymoquinone sec^−1^), the area of the cell monolayer (cm^2^), the volume of the apical compartment (cm^3^), and the initial concentration in the apical compartment (mg thymoquinone), respectively.

The cellular uptake of thymoquinone in Caco‐2 cells was examined using confocal laser scanning microscopy (CLSM) (LSM 700, Carl Zeiss, Germany). The oil was stained with Nile Red (0.001 g mL^−1^) and used as a core solution at coaxial electrospraying. After transport experiments, membranes of well Transwell plates were mounted onto the microscope and cellular uptake of nanoparticles was observed using a CLSM.

### 2.10 Immunomodulatory Potential of BSO‐Loaded Nanoparticles

#### 2.10.1 Cell Growth

Murine macrophage cell line J774.2 cells (ATCC) were utilized in our studies. For their proper growth, media containing 10% fetal bovine serum and Roswell Park Memorial Institute (RPMI 1640) medium supplemented with 1% antibiotics (100 μg cm^−3^ penicillin and 100 μg cm^−3^ streptomycin) was used (Ayaz et al. 2021; Yüzer et al. 2019).

#### 2.10.2 BSO‐Loaded Nanoparticles Treatment of the Macrophages

Cell concentration was 10^6^ cells well^−1^ in 1 mL in each well of the 24 well plates. After the overnight resting in a 37°C 5% CO_2_ humidified incubator, the samples were treated with 1, 5, 10, and 20 μg mL^−1^ of thymoquinone and its derivatives (compound 1: BSO, black seed oil; compound 2: BSO‐oj, orange juice enriched with nonencapsulated BSO; compound 3: NPs, nanoparticles; compound 4: NPS‐oj, orange juice enriched with nanoparticles) with or without 1 μg mL^−1^ of LPS. The cells were treated for 24 h. In the control group, the cells were untreated; in the negative control group, they were treated with 10 μg mL^−1^ of salicylic acid. The supernatants were collected for cytokine analysis by ELISA, and cell viability was assessed by counting cells stained with Trypan blue. Afterwards, the cells were permeabilized and treated with the antibodies for phosphorylated p38 and PI3K levels (Ayaz et al. 2021; Yüzer et al. 2019).

#### 2.10.3 TNFα, IL12p40, GMCSF and IL6 ELISAs

For each ELISA analysis, the guidelines of the manufacturer were strictly followed (BD Biosciences, CA, USA); in this way, we determined TNFα, IL12p40, GMCSF, and IL6 cytokine production levels by the macrophages (Ayaz et al. 2021; Yüzer et al. 2019).

#### 2.10.4 Flow Cytometry Analysis for the Intracellular Staining of the Macrophages

Active phosphorylated PI3K and p38 levels in macrophages were measured using flow cytometry, employing the aforementioned experimental setup. Cell fixing was done by BD Fix Buffer I (557870) and the BD protocols' procedures were followed. After permeabilizing the cells by BD PERM Buffer III (558050), intracellular staining of the cells was done with PE Mouse anti‐PI3K (Invitrogen) and BD PE Mouse anti‐p38 (Invitrogen) by applying the steps specified in the BD protocol. The sample analysis was done by BD FACS ARIA III to determine the phosphorylated PI3K and p38 levels (Ayaz et al. 2021; Yüzer et al. 2019).

### 2.11 Statistical Analysis

Response surface methodology (RSM) was employed to investigate how independent variables affect dependent variables such as the encapsulation efficiency and loading capacity of thymoquinone. The model's adequacy was evaluated by determining the coefficient of determination (*R* ^2^), adjusted *R* ^2^ and coefficient of variation (*CV*). Analysis of variance (ANOVA) was performed to assess the statistical significance of the terms in the regression equation (Altan and Çayır 2020). The differences between the results were analyzed using Duncan's multiple range test in SPSS 16.0 statistical software (SPSS Inc., Chicago, IL, USA). All analyses were performed in at least three replicates. The in vitro data for the macrophage studies were evaluated by Student's t test. Graph Pad Prism version 5 was used to draw the graphs and apply the statistical analysis on the data sets (Ayaz et al. 2021; Yüzer et al. 2019).

## 3 Results and Discussion

### 3.1 EE and LC of Thymoquinone

**FIGURE 1:**
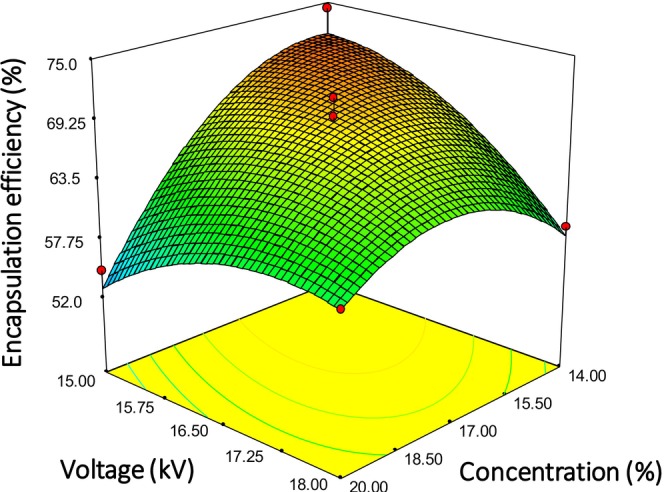
Effect of applied voltage and concentration of zein solution on the encapsulation efficiency at a distance of 15.5 cm and flow rate of 0.55 mL/h.

The previous study shows that the BSO encapsulation within zein nanoparticles can be successfully achieved through coaxial electrospraying. BSO‐loaded zein nanoparticles were obtained with a nano‐size, homogeneous, smooth, and spherical morphology (Atay and Altan 2021). EE provides insight into how well the chosen encapsulation approach can trap and retain the active compound within the nanoparticles. Therefore, EE and LC were used to assess the success of encapsulating thymoquinone by coaxial electrospraying. EE indicates the amount of thymoquinone actually encapsulated, while LC represents the amount of thymoquinone in a certain amount of nanoparticles. EE and LC of thymoquinone‐loaded nanoparticles varied from 49.43% ± 1.4 to 74.38% ± 2.6% and 1.31% ± 0.2 to 12.99% ± 3.6%, respectively. These findings are also in agreement with earlier reports that detail the EE and LC of thymoquinone‐loaded nanoparticles (Alam et al. 2012; Ansar et al. 2020; Rahat et al. 2021). Tables S2 and S3 summarize the analysis of variance (ANOVA) results for the effect of variables on EE and LC of thymoquinone. ANOVA results showed that the flow rate of the core solution, zein solution concentration, and voltage significantly affected the EE and LC (*p* < 0.05), but the effect of the change in distance between the needle and collector was not significant. An increase in the voltage and the core solution flow rate resulted in a decrease in EE and LC (Figure 1). The applied voltage between the needle tip and collector in the coaxial electrospraying technique plays a significant role in the formation of a stable cone‐jet. The Taylor cone formed by both the core and shell solution liquids typically exhibits a symmetric structure with a thin jet at the top in the stable cone‐jet mode of coaxial electrospraying. This thin liquid jet is subject to both electrical and hydrodynamic forces, which cause the jet to break into smaller droplets or particles. The core and shell solution flow rates also significantly influence cone‐jet stability. Therefore, the decrease in EE and LC was attributed to the unstable cone‐jet formation with increasing the flow rate of the core solution and the voltage. In our previous study, we obtained a larger particle diameter with increasing the voltage at high core solution flow rate and zein concentration for the BSO‐loaded nanoparticles. This study also revealed that particle coalescence occurred as a result of incomplete solvent volatilization under the given process conditions. These changes in morphological structure were considered an indication of the polymer jet instability. The high encapsulation efficiency was reported for the particles in spherical and uniform size distribution (Atay and Altan 2021). The concentration of the shell solution is a substantial parameter for achieving the desired hard shell around core materials. The EE of thymoquinone decreased when the zein concentration in the shell solution was increased (Figure 1). The increase in viscosity with increasing concentration may prevent obtaining a steady cone‐jet, thus resulting in a low EE value. The highest EE and LC of thymoquinone were found at the flow rate of the core solution 0.55 mL h^−1^, the applied voltage at 15 kV, 15.5 cm distance, and 14% zein concentration in shell solution (B1), whereas the lowest EE and LC values were found for nanoparticles produced at a flow rate of 0.70 mL h^−1^, a distance of 15.5 cm, an applied voltage of 16.5 kV, and 20% zein concentration in shell solution (W1).

### 3.2 Thymoquinone Stability in BSO‐Loaded Nanoparticles During Storage

**FIGURE 2:**
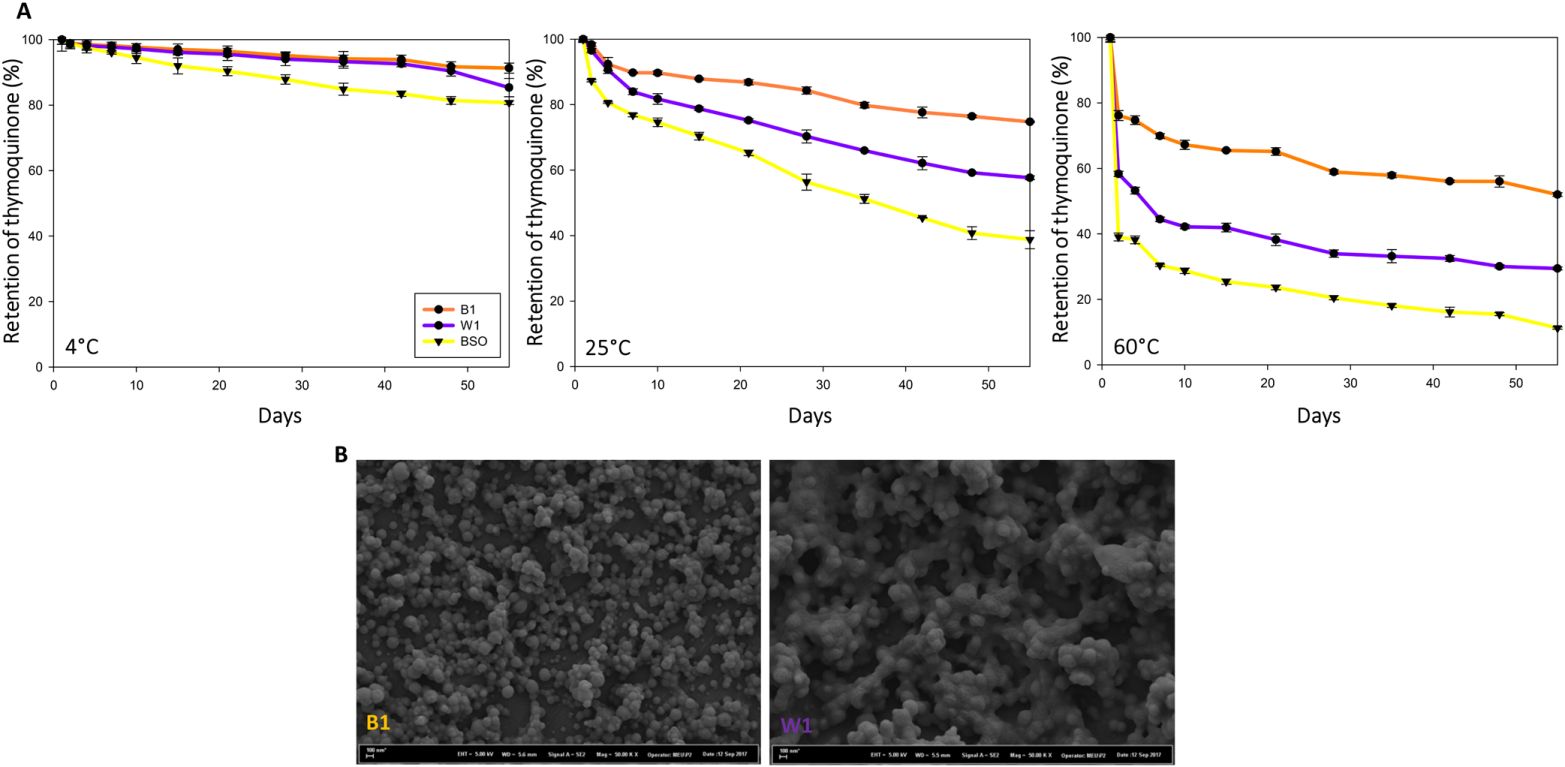
(A) Effect of storage on thymoquinone retention in different samples over 55 days at 4°C, 25°C, and 60°C; B1: Nanoparticles with high encapsulation efficiency, W1: Nanoparticles with low encapsulation efficiency, BSO: Nonencapsulated black seed oil, (B) FE‐SEM images of nanoparticles (B1 and W1).

One of the most critical factors in the encapsulation of bioactive compounds is the capability of the wall material to retain the volatile compounds during storage. BSO‐loaded nanoparticles, coded B1 and W1, were reproduced following the experimental parameters of run 8 and run 19 to assess their storage stability. Samples B1 and W1 were selected for producing BSO‐loaded nanoparticles due to their relatively high and low encapsulation efficiency and loading capacity, respectively. Figure 2A shows the thymoquinone retention in nanoparticles and nonencapsulated oil after 55 days of storage at 4°C, 25°C, and 60°C. The thymoquinone in the nonencapsulated oil decreased by around 60% at 25°C and over 80% at 60°C. In contrast, the retention of thymoquinone at 25°C in nanoparticles coded B1 and W1 was approximately 80% and 60%, respectively, indicating the effective protective effects of encapsulation. Compared to the retention of thymoquinone in nanoparticles coded B1 and W1, thymoquinone in nanoparticles coded B1 exhibited better storage stability. This result concurs with the results of the encapsulation efficiency, showing a more complete encapsulation of BSO and thus the highest retention of thymoquinone during storage. The nanoparticles coded B1 are homogenous and regular, which directly influences the retention of the encapsulated bioactive compound (Figure 2B). On the other hand, the nanoparticles coded W1 exhibited low thymoquinone retention due to their low encapsulation efficiency, which indicates incomplete encapsulation of thymoquinone. The higher release may be due to the irregular and nonspherical shapes of the nanoparticles (Atay and Altan 2021). The results indicate that the retention of thymoquinone during long‐term storage is affected by the encapsulation of oil and the morphological changes of nanoparticles. The retention of thymoquinone was lowest in samples stored at 60°C, where increasing temperature increased the release rate and diffusion of the essential oil (Li and Lu 2016). However, the nanoparticles coded B1 exhibited effective retention of thymoquinone even at high temperatures. This result was in agreement with Abedi et al. (2021) who reported that the encapsulation of *N. sativa* seeds oil in microparticles significantly reduced the percentage loss of thymoquinone. According to their findings, thymoquinone content decreased by 23% in spray‐dried particles using a mixture of maltodextrin and modified starch wall materials after storage at 20°C for 30 days. Karaman (2020) reported 52.63% loss of thymoquinone in BSO‐loaded yeast microcapsules stored at 65°C for 8 days. In our study, nanoparticles stored at 60°C for 55 days exhibited 48% loss of thymoquinone, whereas the loss of thymoquinone in nonencapsulated BSO was 88.75%. These results showed that nanoencapsulation of BSO with zein using electrospraying significantly enhanced the stability of thymoquinone.

### 3.3 Bioaccessibility of Thymoquinone

**FIGURE 3:**
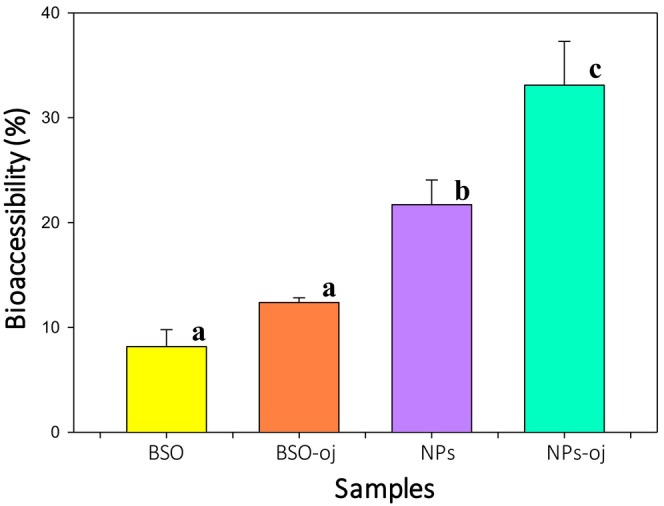
Bioaccessibility of thymoquinone in in vitro gastrointestinal conditions (BSO, black seed oil; BSO‐oj, orange juice enriched with nonencapsulated black seed oil; NPs, nanoparticles; NPS‐oj, orange juice enriched with nanoparticles).

Bioaccessibility is a prerequisite for bioavailability, and in vitro experiments that mimic physiological conditions can provide valuable information about the bioavailability of bioactive compounds in various food systems (Mackie et al. 2020). It refers to the proportion of a nutrient that is released from the food matrix during digestion and becomes accessible for uptake by the intestinal epithelium, representing a critical step prior to systemic absorption (Shi et al. 2024). Bioactive compounds such as thymoquinone have poor water solubility and are unstable during storage or digestion conditions, so they are not readily bioaccessible. Thus, increasing the bioaccessibility of thymoquinone through nanoencapsulation is an important step in improving its bioactivity. To investigate the effect of encapsulation and food matrix on the bioaccessibility of thymoquinone, the total thymoquinone content was measured after in vitro GI digestion of nanoparticles, orange juice fortified with nanoparticles, nonencapsulated oil, and orange juice fortified with nonencapsulated oil. Figure 3 presents the bioaccessibility (%) of the thymoquinone after in vitro GI digestion. The bioaccessibility of thymoquinone from nonencapsulated oil and nanoparticles with high EE was 8.2% and 21.7%, respectively. The encapsulation of oil significantly increased the bioaccessibility of thymoquinone by 2.6‐fold compared to the nonencapsulated oil. This may be attributed to the fact that nanoencapsulation with zein protects thymoquinone under gastric conditions and allows its controlled release in the intestinal fluid (Mahalakshmi et al. 2020). Our previous study showed a decrease in the released amount of thymoquinone from free BSO during the intestinal phase of digestion, suggesting that thymoquinone undergoes degradation under these conditions (Atay and Altan 2021). The degradation of thymoquinone in free oil may occur as a result of pH changes and interactions with components of the digestive fluids, including enzymes and electrolytes (Finos et al. 2024). Rodríguez‐Félix et al. (2019) reported that quercetin encapsulated in nanoparticles exhibited greater gastrointestinal stability and bioaccessibility compared to free quercetin. Smaller particle size with larger surface area promotes more effective dissolution of nanoparticles, increasing the release of thymoquinone and improving its bioaccessibility. During lipid digestion, the lipid digestion products such as free fatty acids and monoacylglycerols interact with bile salts and phospholipids to form mixed micelles that solubilized the released thymoquinone (Rein et al. 2013). Similar results were observed by Ardhi et al. (2025) where encapsulation of thymoquinone in nanostructured lipid carriers improved its solubility and bioaccessibility during gastrointestinal digestion. Stefani et al. (2019) investigated the in vitro digestion behavior of linseed oil loaded in chia seed mucilage nanoparticles and reported a bioaccessibility of 12.80% after digestion. The study showed that nanoencapsulation promoted a good bioaccessibility of linseed oil and enhanced the stability of the oil during in vitro digestion.

The highest thymoquinone bioaccessibility was observed in orange juice fortified with nanoparticles, reaching 33.1%, which was significantly higher than the 12.4% observed in juice fortified with nonencapsulated oil (Figure 3). However, there was no significant difference between the in vitro GI bioaccessibility of thymoquinone from nonencapsulated oil and orange juice fortified with nonencapsulated oil. The in vitro GI bioaccessibility of nanoencapsulated thymoquinone significantly increased in the presence of orange juice. This improvement may be attributed to the protective effect of nanoencapsulation and the enhanced solubility and stability of thymoquinone during digestion. In addition, orange juice components such as citric acid and ascorbic acid can influence lipid digestion and affect mixed micelle formation, thereby enhancing the solubilization and bioaccessibility of thymoquinone (Speranza et al. 2017; Gonçalves et al. 2023). Similarly, Gonçalves et al. (2023) reported an increased bioaccessibility of curcumin in a model beverage fortified with solid lipid nanoparticles compared to a beverage containing free curcumin. Ruiz‐Rico et al. (2017) investigated the bioaccessibility of the encapsulated folic acid using mesoporous silica particles (MSPs) during the simulated digestion of apple and orange juices. They observed a faster release of folic acid in orange juice, whereas a progressive release occurred in apple juice, suggesting that the juice matrix can influence the release and bioaccessibility of encapsulated bioactives.

### 3.4 Transport Assay to Estimate Intestinal Absorption of Thymoquinone

**FIGURE 4:**
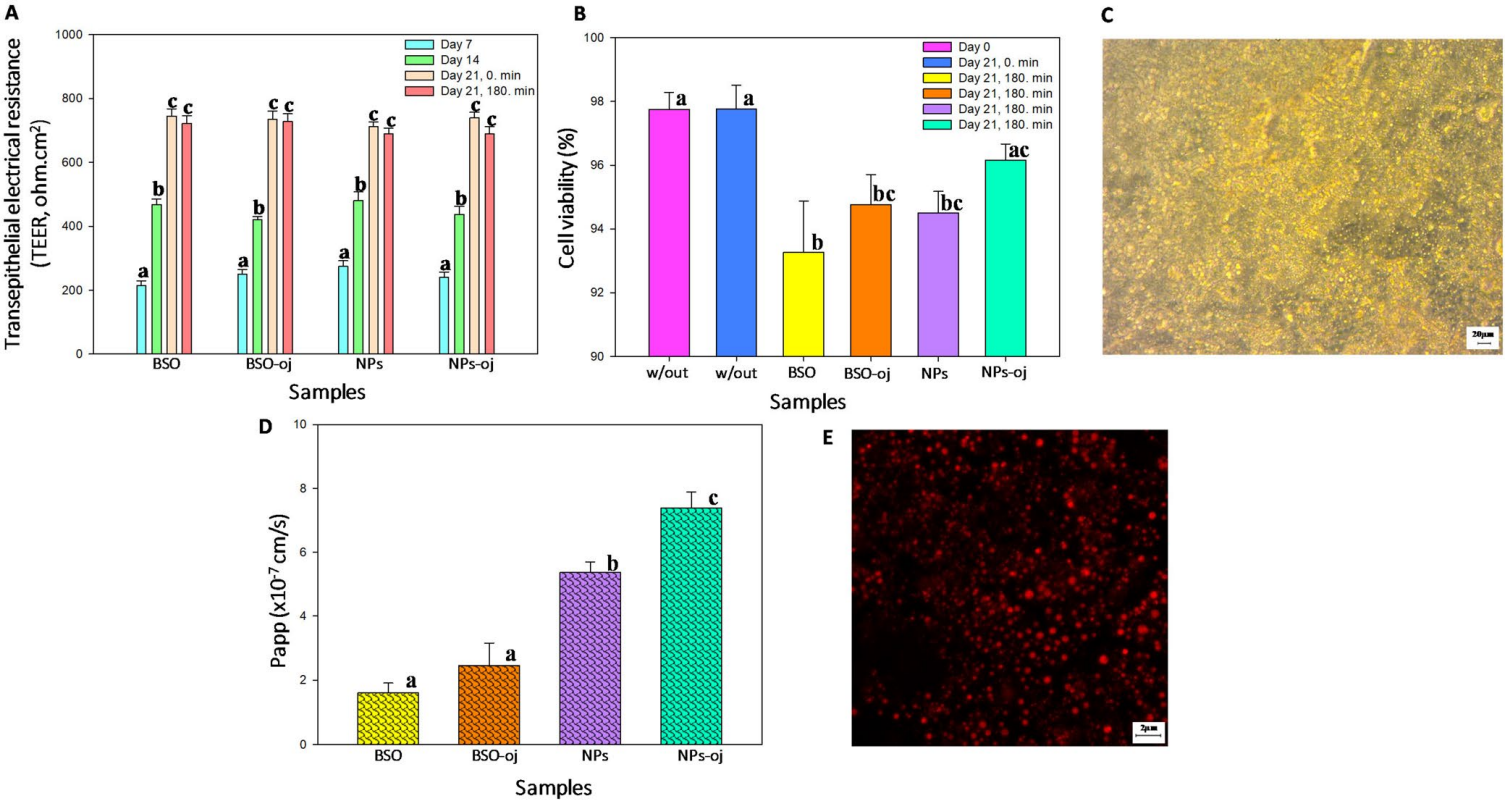
(A) The transepithelial electrical resistance (TEER) values up to 21 days, (B) the viability of Caco‐2 cell lines during transport assay, (C) images of Caco‐2 cells at the end of the transport assays using nanoparticles by inverted microscope (40×), (D) transport assays across Caco‐2 monolayers BSO, black seed oil; BSO‐oj, orange juice enriched with nonencapsulated black seed oil; NPs, nanoparticles; NPS‐oj, orange juice enriched with nanoparticles, (E) image of Caco‐2 cells at the end of the transport assays using nanoparticles by confocal microscope.

In vitro bioaccessibility studies revealed how much of the thymoquinone contained in encapsulated and nonencapsulated samples was available for absorption following GI digestion. Subsequently, the concentration of thymoquinone that was able to pass through the intestinal wall and enter the bloodstream was determined in the same samples. To investigate intestinal absorption, Caco‐2 cell monolayers were employed. Additionally, only the digested extracts were used to mimic the natural human absorption process in the intestine. TEER values (< 25%), dye leakage (< 2%), and cell viability were tested to confirm the integrity of the Caco‐2 cell monolayer before conducting the transport assays. From Figure 4A, the initial TEER values were above 700 Ω cm^−2^, indicating that the Caco‐2 cell monolayers were successfully constructed and that the monolayers had a good level of integrity for the transport assays. The cell viability values were higher than 95% for all samples at the initial stage of the assay, confirming that the monolayers were suitable for further testing (Figure 4B). Additionally, the cells largely maintained their viability at the end of the transport assay. As expected, the zein nanoparticles and oil did not have a significant (*p >* 0.05) toxic effect on the Caco‐2 cell viability. In literature, in vitro cell cytotoxicity assays suggested that zein nano carriers were nontoxic and biocompatible (Nunes et al. 2020). Transepithelial electrical resistance results show no significant decrease in TEER values (< 25%) at the end of the transport assay. Inverted microscope images confirmed that the Caco‐2 cells maintained their structural integrity during the assay with nanoparticles, thereby validating the robustness of the experimental procedure (Figure 4C). These results indicate that the nanoparticles formulated with natural biopolymers are non‐toxic and biocompatible. Moreover, the encapsulation process and fortification of orange juice supported cell viability. Therefore, the zein nanoparticles developed in this study show promise as a safe and effective carrier for thymoquinone delivery.

Transport assay results were expressed simply by determining from apical to basolateral apparent permeability values (*P*_app_, expressed in cm s^−1^). Concerning encapsulated and nonencapsulated BSO samples, the *P*_app_ for thymoquinone ranged between 2.10 × 10^−7^ and 5.76 × 10^−7^ cm s^−1^ (Figure 4D). The *P*_app_ value of thymoquinone in nanoparticles (NPs) was 2.74‐fold higher than that of thymoquinone in nonencapsulated oil (BSO). The cellular uptake of thymoquinone was demonstrated by staining the oil within the nanoparticles with Nile red (Figure 4E). According to literature, the *P*_app_ values of most compounds fall within the range of 1.0 × 10^−7^ cm s^−1^ to 1.0 × 10^−5^ cm s^−1^, corresponding to poor and high permeability, respectively (Bhushani et al. 2016; Silva et al. 2019). In the present study, the low apparent *P*_app_ of thymoquinone showed its limited transepithelial absorption when delivered in free BSO. However, encapsulation of thymoquinone significantly increased its transepithelial absorption (Figure 4D). These findings align with those of Lodovichi et al. (2022), who reported higher *P*_app_ values for thymoquinone encapsulated in polymeric micelles compared to its free thymoquinone. They found a *P*_app_ value of 1.5 × 10^−5^ cm s^−1^ across the Caco‐2 cell model after 2 h of incubation, which was higher than the value obtained in the present study. This difference may be due to the use of both solubilizing agents and penetration enhancers which facilitated greater passive diffusion of thymoquinone across the membrane. Deng et al. (2024) also reported that the apparent permeability of curcumin‐loaded nanoparticles was greater than that of free curcumin. In another study, Tian et al. (2024) demonstrated that soy isoflavone nanoparticles were more readily internalized by intestinal cells compared to free soy isoflavones. These results suggest that encapsulation effectively enhances the permeability of active compounds. Such behavior may result from the ability of nanoparticles to undergo cellular uptake through endocytosis, followed by their exocytotic transport to the basolateral side of Caco‐2 monolayers. The encapsulation of bioactive compounds within wall materials resulted in the protection of bioactives during digestion and made them available for intestinal absorption through the Caco‐2 cells. In addition, it has been reported that small‐sized nanoparticles can enhance the transport of bioactive compounds across Caco‐2 cell monolayers, owing to their advantages such as low steric hindrance and rapid interaction with cell membranes (Yuan et al. 2024; Walia and Chen 2020; Zhang et al. 2015). According to Jayan et al. (2019), the mucoadhesive characteristics of zein improve permeability by extending its retention on the intestinal epithelium, thereby enhancing the concentration gradient required for effective absorption of the active molecule.

The apparent permeability of thymoquinone in orange juice fortified with nanoparticles was determined to be 7.38 × 10^−7^ cm s^−1^. This was the highest *P*_app_ value observed among the samples, indicating enhanced diffusion of thymoquinone across Caco‐2 cell layers. This improvement may be attributed to the role of orange juice as an active food matrix, which likely enhanced the solubility and micellar incorporation of thymoquinone. These are essential for improving its bioaccessibility and subsequent intestinal absorption (Tan and McClements 2021).

The transport efficiency results further confirmed the enhanced performance of nanoencapsulation. Specifically, thymoquinone‐loaded nanoparticles significantly improved transport efficiency compared to nonencapsulated oil (*p* < 0.05) (Table 1). These findings highlight that encapsulating black seed oil (BSO) in nanoparticles is an effective strategy to enhance cellular uptake and transport of thymoquinone.

**TABLE 1:** Apical and basolateral side recoveries and transport efficiencies of thymoquinone from BSO, black seed oil; BSO‐oj, orange juice enriched with unencapsulated black seed oil; NPs, nanoparticles; NPS‐oj, orange juice enriched with nanoparticles.

Digested sample	*t* = 180 min		
	Apical recovery (%)^A^	Basolateral recovery (%)^B^	Transport efficiency^C^ ^s^
BSO	44.71 ± 3.9^a*^	1.70 ± 0.5^a^	0.038 ± 0.002^a^
BSO‐oj	44.72 ± 4.5^a^	1.70 ± 0.4^a^	0.038 ± 0.001^a^
NPs	63.40 ± 3.2^b^	2.62 ± 0.9^b^	0.041 ± 0.006^b^
NPs‐oj	64.11 ± 5.3^c^	2.68 ± 0.1^c^	0.042 ± 0.001^b^

*Note:* *Within each column, values with different superscript letters are significantly different (*p* < 0.05).

^A^ Apical recovery = (thymoquinone concentration after transport)/(thymoquinone concentration at 0 h of incubation) × 100.

^B^ Basolateral recovery = (thymoquinone concentration after transport)/(thymoquinone concentration at 0 h of incubation) × 100.

^C^ Transport efficiency = (basolateral side recovery, %)/(apical side recovery, %).

### 3.5 Immunomodulatory Potential of Thymoquinone

**FIGURE 5:**
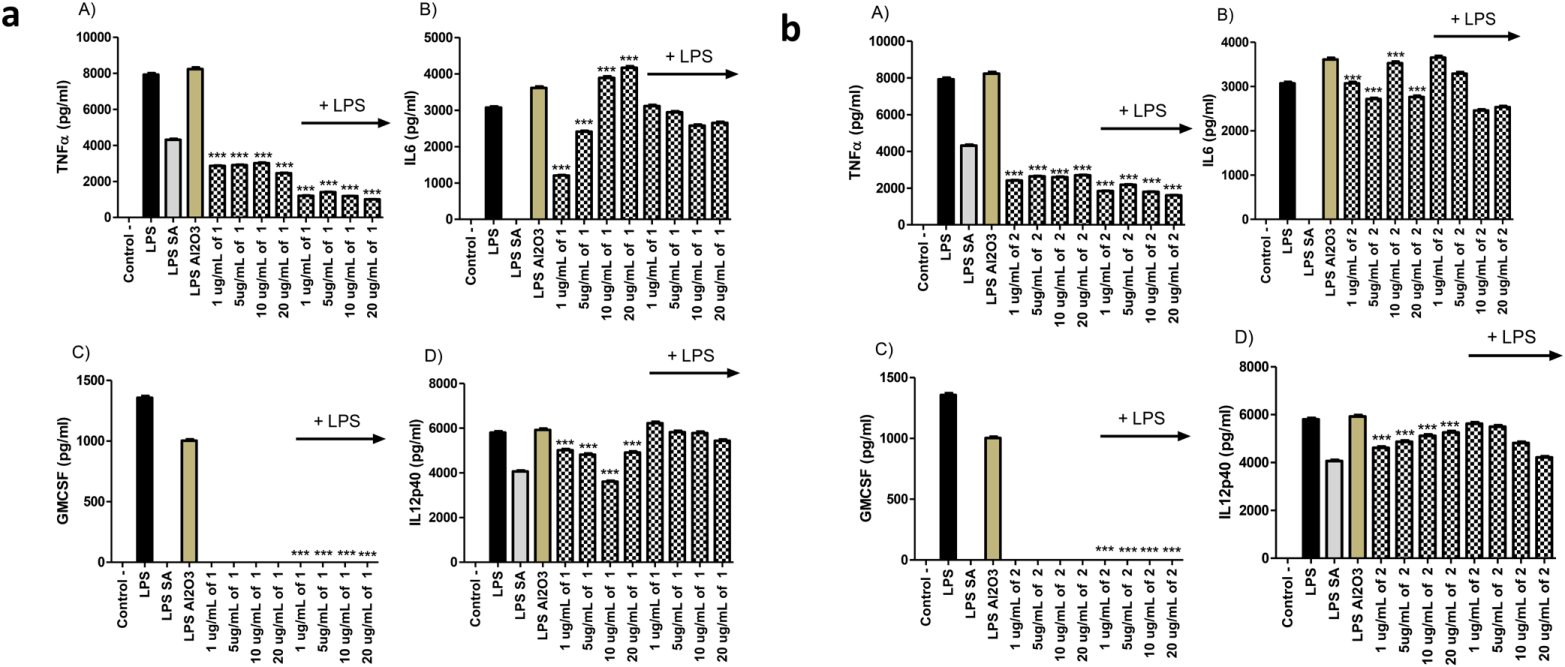
TNF, IL6, GMCSF, and IL12p40 ELISA for the supernatants of the unstimulated and 1 μg mL^−1^ of LPS stimulated cells after 24 h of incubation. 1, 5, 10 and 20 μg mL^−1^ of compound was put together with 1 μg mL^−1^ of LPS into the respective wells. 1 × 10^6^ cells mL^−1^ of J774.2 mammalian macrophages were incubated overnight and then stimulated for 24 h. 10 μg mL^−1^ of salicylic acid was used as control. Student's *t* test statistical analysis was conducted on each data set consisting of three biologically independent experiments by GraphPad Prism Version 5 (*p < 0.001, **p < 0.0005, ***p < 0.0001, *N* = 3) (a) compound 1 (b) compound 2.

**FIGURE 6:**
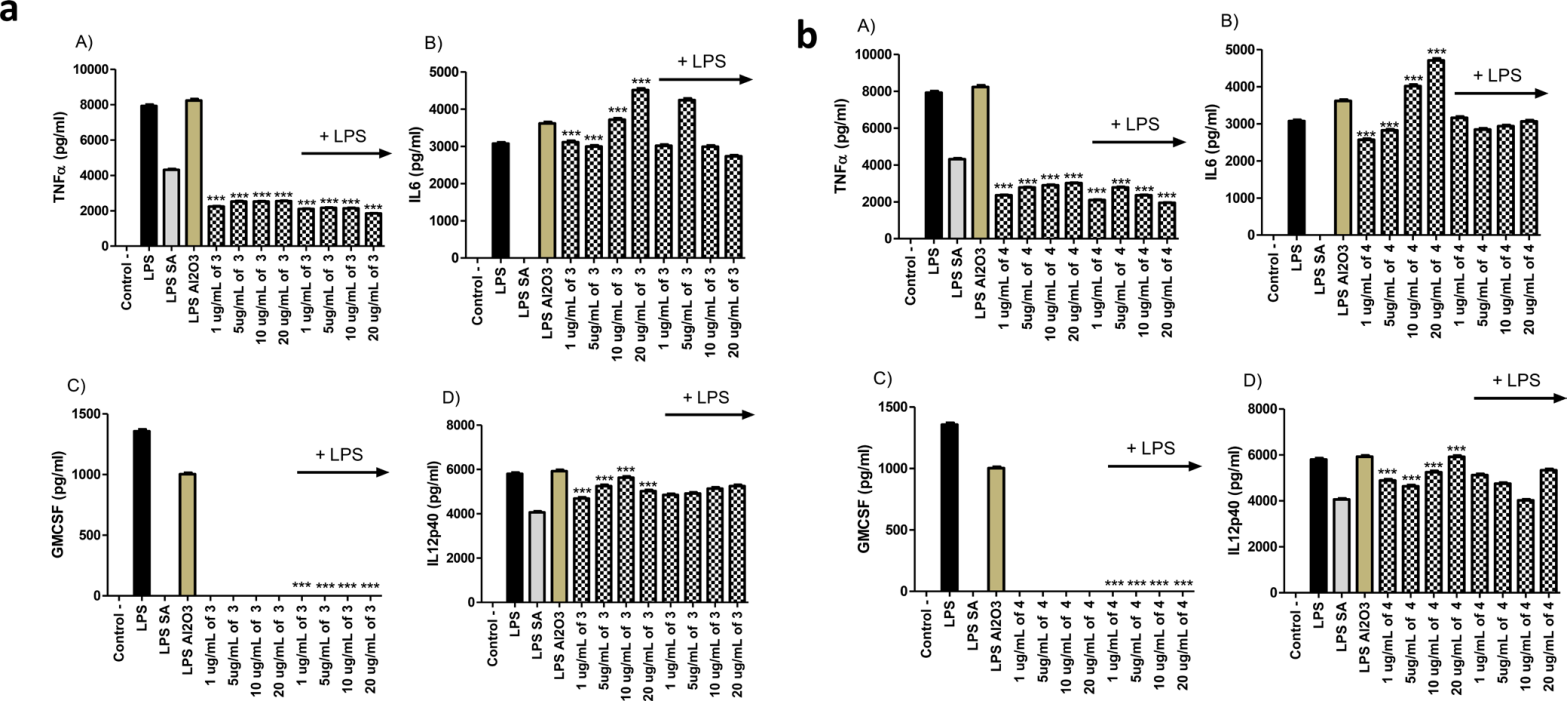
TNF, IL6, GMCSF, and IL12p40 ELISA for the supernatants of the unstimulated and 1 μg mL^−1^ of LPS stimulated cells after 24 h of incubation. 1, 5, 10, and 20 μg mL^−1^ of compound was put together with 1 μg mL^−1^ of LPS into the respective wells. 1 × 10^6^ cells mL^−1^ of J774.2 mammalian macrophages were incubated overnight and then stimulated for 24 h. 10 μg mL^−1^ of salicylic acid was used as control. Student's *t* test statistical analysis was conducted on each data set consisting of three biologically independent experiments by GraphPad Prism Version 5 (*p < 0.001, **p < 0.0005, ***p < 0.0001, *N* = 3) (a) compound 3, (b) compound 4.

**FIGURE 7:**
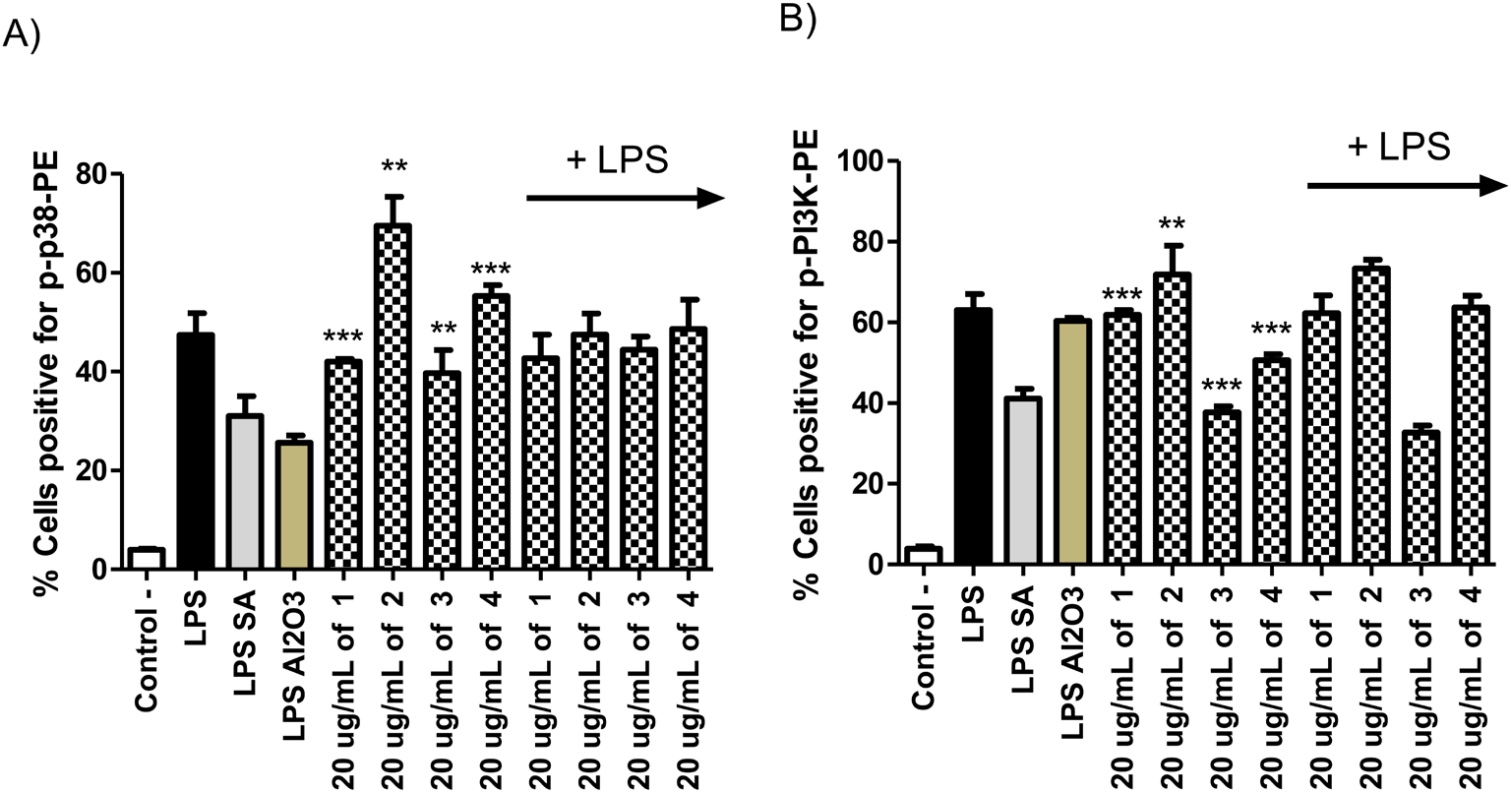
J774.2 cells were incubated in 10^6^ cells per well in 1 mL concentration in 24 well plates for overnight resting in a 37°C 5% CO_2_ humidified incubator. 20 μg mL^−1^ of each compound was used with 1 μg mL^−1^ LPS for 24 h cell activation. There was no chemical added control group, 10 μg mL^−1^ of salicylic acid added negative control group together with 1 μg mL^−1^ of LPS, there was only 1 μg mL^−1^ of LPS in the positive control group. After the incubation the cells were utilized to determine active phosphorylated p38 (A) and PI3K (B) levels in macrophages. Student's *t* test statistical analysis was conducted on each data set consisting of three biologically independent experiments by GraphPad Prism Version 5 (*p < 0.001, **p < 0.0005, ***p < 0.0001, *N* = 3).

Immune system regulation is important for the therapeutic utilization of encapsulated thymoquinone. In our experimental setup, we compared the activities of the nonencapsulated and encapsulated thymoquinone for their immunomodulatory activities on the mammalian macrophages. First of all, when the cell viabilities were compared, our compounds did not have a negative impact on the cell viability of the macrophages (Data not shown). When the immunostimulatory activities of the thymoquinone and encapsulated thymoquinone were compared, they activated the macrophages to produce pro‐inflammatory cytokines TNFα, IL6, GMCSF, and IL12p40 to substantial levels (Figures 5 and 6). This suggests their potential utilization as adjuvant candidates. When they were given together with LPS, though, they had exact opposite activity and exerted an anti‐inflammatory activity by substantially blocking the production of these pro‐inflammatory cytokines significantly compared to only LPS treated control groups (Figures 5 and 6). These results are in line with the results of the previous studies. There have been studies in the literature supporting the immunomodulatory and anti‐inflammatory activity of thymoquinone. Most of the studies primarily focus on the *Nigella sativa* seed extracts that are rich in thymoquinone content. When these extracts were utilized, the studies suggest that there was a decrease in pro‐inflammatory cytokine production levels (Hanieh et al. 2018; Koçyiğit and Güler 2021; Majdalawieh and Fayyad 2015; Figlon et al. 2023; Koshak et al. 2018). For the first time to our knowledge, in our study, we are comparing these activities in depth with intracellular signaling components, and also we are using nanoencapsulated thymoquinone derivatives to compare the functional differences when the thymoquinone was derivatized for higher bioaccessibility. When the intracellular pathways were analyzed by flow cytometry, our compounds had a substantial effect on the phosphorylated (active) levels of p38 and PI3K (Figure 7). These pathways have been linked with inflammatory responses, and thymoquinone and encapsulated thymoquinone derivatives exerted their activities through the activation or suppression of these pathways (Bewley et al. 2016). Previous studies suggest the involvement of NFκB and COX pathways in the immunomodulatory effect of thymoquinone (Yazdi et al. 2018; Majdalawieh and Fayyad 2015; Figlon et al. 2023; Koçyiğit and Güler 2021; Koshak et al. 2018). Our results suggest that p38 MAPK and PI3K pathways also play a major role in the activity of the thymoquinone and encapsulated thymoquinone (Figure 7).

## 4 Conclusions

The nanoencapsulation of BSO with zein using electrospraying significantly enhanced the stability of thymoquinone. The loss of thymoquinone in nanoparticles with high encapsulation efficiency was 8.7%, 25.3%, and 48%, whereas the loss in free BSO was 19.3%, 61.2%, and 88.7% during 55 days of storage at 4°C, 25°C, and 60°C, respectively. The incorporation of nanoparticles into orange juice resulted in a significantly higher thymoquinone bioaccessibility (33.1%) compared to juice fortified with nonencapsulated oil (12.4%). A 3.5‐fold increase in thymoquinone's apparent permeability was achieved through nanoencapsulation of BSO and the use of orange juice as a delivery medium. Moreover, thymoquinone nanoparticles triggered macrophages to release high levels of pro‐inflammatory cytokines, including TNFα, IL6, GMCSF, and IL12p40. Future studies could investigate thymoquinone's bioaccessibility and bioavailability in different food matrices, facilitating the development of functional foods fortified with BSO‐loaded nanoparticles.

## Author Contributions

**Elif Atay:** formal analysis (equal), investigation (equal), methodology (equal), writing – original draft (equal), writing – review and editing (equal). **Aylin Altan:** conceptualization (lead), formal analysis (equal), investigation (equal), methodology (equal), project administration (lead), supervision (lead), visualization (lead), writing – original draft (equal), writing – review and editing (equal). **Derya Yetkin:** formal analysis (equal), methodology (equal), writing – original draft (equal), writing – review and editing (equal). **Furkan Ayaz:** formal analysis (equal), methodology (equal), writing – original draft (equal), writing – review and editing (equal).

## Conflicts of Interest

The authors declare no conflicts of interest.

## Supporting information


Data S1

## Data Availability

The data that support the findings of this study are available from the corresponding author upon reasonable request.
