# Supplementary material for: Coaxial Electrospraying for Black Seed Oil Nanoencapsulation: Improved Thymoquinone Stability and Bioactivity
Source: Food Sci Nutr. 2025 Sep 15;13(7):e70622. doi: 10.1002/fsn3.70622 (PMC12434836; doi:10.1002/fsn3.70622)
Supplement: Supplementary file 1 — Data S1 [file FSN3-13-e70622-s001.docx]

**Coaxial electrospraying for black seed oil nanoencapsulation: improved thymoquinone stability and bioactivity**

Elif Atay^a^, Aylin Altan^a^*, Derya Yetkin^b^, Furkan Ayaz^c*^

^a^Department of Food Engineering, Mersin University, Mersin, 33343, Türkiye

^b^Department of Histology and Embryology, Mersin University, Mersin, 33343, Türkiye

^c^Department of Molecular Biology and Genetics, Faculty of Engineering and Natural Sciences, Istinye University, Istanbul, 34010, Türkiye

*Corresponding Authors: [aaltan@mersin.edu.tr](mailto:aaltan@mersin.edu.tr), fayaz@biruni.edu.tr

**Running title:**

**Coaxial electrospraying for thymoquinone delivery**

**Table S1.** Electrospraying experimental design with coded and actual variable levels

|  | Coded levels | | | |  | Actual levels | | | |
| --- | --- | --- | --- | --- | --- | --- | --- | --- | --- |
| Run | A B C D | | | |  | Concentration  (%) | Voltage  (kV) | Distance (cm) | Flow rate of core solution  (mL/h) |
| 1 | 0 | 1 | -1 | 0 |  | 17 | 18.0 | 14.0 | 0.55 |
| 2 | -1 | 0 | 1 | 0 |  | 14 | 16.5 | 17.0 | 0.55 |
| 3 | 0 | 0 | -1 | -1 |  | 17 | 16.5 | 14.0 | 0.40 |
| 4 | -1 | 0 | 0 | 1 |  | 14 | 16.5 | 15.5 | 0.70 |
| 5 | -1 | 1 | 0 | 0 |  | 14 | 18.0 | 15.5 | 0.55 |
| 6 | 0 | -1 | 1 | 0 |  | 17 | 15.0 | 17.0 | 0.55 |
| 7 | 0 | 0 | 0 | 0 |  | 17 | 16.5 | 15.5 | 0.55 |
| 8 | -1 | -1 | 0 | 0 |  | 14 | 15.0 | 15.5 | 0.55 |
| 9 | 0 | 1 | 0 | -1 |  | 17 | 18.0 | 15.5 | 0.40 |
| 10 | 1 | -1 | 0 | 0 |  | 20 | 15.0 | 15.5 | 0.55 |
| 11 | 0 | 0 | 0 | 0 |  | 17 | 16.5 | 15.5 | 0.55 |
| 12 | 1 | 0 | -1 | 0 |  | 20 | 16.5 | 14.0 | 0.55 |
| 13 | 0 | -1 | 0 | -1 |  | 17 | 15.0 | 15.5 | 0.40 |
| 14 | 1 | 0 | 1 | 0 |  | 20 | 16.5 | 17.0 | 0.55 |
| 15 | 0 | 0 | -1 | 1 |  | 17 | 16.5 | 14.0 | 0.70 |
| 16 | 0 | 0 | 0 | 0 |  | 17 | 16.5 | 15.5 | 0.55 |
| 17 | 1 | 0 | 0 | -1 |  | 20 | 16.5 | 15.5 | 0.40 |
| 18 | 0 | 0 | 0 | 0 |  | 17 | 16.5 | 15.5 | 0.55 |
| 19 | 1 | 0 | 0 | 1 |  | 20 | 16.5 | 15.5 | 0.70 |
| 20 | 0 | 0 | 1 | 1 |  | 17 | 16.5 | 17.0 | 0.70 |
| 21 | -1 | 0 | -1 | 0 |  | 14 | 16.5 | 14.0 | 0.55 |
| 22 | 0 | 0 | 0 | 0 |  | 17 | 16.5 | 15.5 | 0.55 |
| 23 | 0 | 0 | 1 | -1 |  | 17 | 16.5 | 17.0 | 0.40 |
| 24 | 0 | 1 | 0 | 1 |  | 17 | 18.0 | 15.5 | 0.70 |
| 25 | 0 | 1 | 1 | 0 |  | 17 | 18.0 | 17.0 | 0.55 |
| 26 | -1 | 0 | 0 | -1 |  | 14 | 16.5 | 15.5 | 0.40 |
| 27 | 0 | -1 | -1 | 0 |  | 17 | 15.0 | 14.0 | 0.55 |
| 28 | 0 | -1 | 0 | 1 |  | 17 | 15.0 | 15.5 | 0.70 |
| 29 | 1 | 1 | 0 | 0 |  | 20 | 18.0 | 15.5 | 0.55 |

**Table S2.** ANOVA results for fitted models of encapsulation efficiency

| **Source** | **Encapsulation efficiency** | | | | |
| --- | --- | --- | --- | --- | --- |
|  | *Sum of squares* | *df* | *Mean square* | *F value* | *p value* |
| **Model** | 1149.19 | 14 | 82.08 | 9.53 | < 0.0001 |
| **A- Shell concentration (%)** | 215.99 | 1 | 215.99 | 25.08 | 0.0002 |
| **B-Voltage (kV);** | 41.17 | 1 | 41.17 | 4.78 | 0.0463 |
| **C-** **Distance (%)** | 0.059 | 1 | 0.059 | 6.876E-003 | 0.9351 |
| **D-Flow rate (mL)** | 279.55 | 1 | 279.55 | 32.46 | < 0.0001 |
| **AB** | 107.93 | 1 | 107.93 | 12.53 | 0.0033 |
| **AC** | 8.63 | 1 | 8.63 | 1.00 | 0.3339 |
| **AD** | 27.34 | 1 | 27.34 | 3.17 | 0.0965 |
| **BC** | 0.65 | 1 | 0.65 | 0.076 | 0.7874 |
| **BD** | 0.042 | 1 | 0.042 | 4.831E-003 | 0.9456 |
| **CD** | 35.08 | 1 | 35.08 | 4.07 | 0.0632 |
| **A^2^** | 173.27 | 1 | 173.27 | 20.12 | 0.0005 |
| **B^2^** | 67.93 | 1 | 67.93 | 7.89 | 0.0140 |
| **C^2^** | 14.90 | 1 | 14.90 | 1.73 | 0.2096 |
| **D^2^** | 319.34 | 1 | 319.34 | 37.08 | < 0.0001 |
| **Residual** | 120.58 | 14 | 8.61 |  |  |
| **Lack of fit** | 110.92 | 10 | 11.09 | 4.59 | 0.0775 |
| **Pure error** | 9.66 | 4 | 2.42 |  |  |
| **Total** | 1269.77 | 28 |  |  |  |
| **Model summary** | | | | | |
| **Standard deviation** | 2.93 | **R^2^ squared** | 0.9050 |  |  |
| **Mean** | 61.90 | **Adjusted R^2^** | 0.8101 |  |  |
| **C.V (%)** | 4.74 | **Adeq Precision** | 10.198 |  |  |
| Encapsulation efficiency=-373.21-9.18A+29.13B+25.57C+408.60D+1.15A*B+0.32A*C+5.80A*D-0.17B*C+0.45B*D-13.16C*D-0.57A^2^-1.43B^2^-0.67C^2^-311.85D^2^ | | | | | |
| *p<0.05 significant, **p<0.01 significant, ns not significant | | | | | |

**Table S3.** ANOVA results for fitted models of loading capacity

| **Source** | **Loading capacity** | | | | |
| --- | --- | --- | --- | --- | --- |
|  | *Sum of squares* | *df* | *Mean square* | *F value* | *p value* |
| **Model** | 149.72 | 14 | 10.69 | 3.85 | 0.0084 |
| **A- Shell concentration (%)** | 18.42 | 1 | 18.42 | 6.63 | 0.0220 |
| **B-Voltage (kV);** | 15.13 | 1 | 15.13 | 5.44 | 0.0351 |
| **C-** **Distance (%)** | 0.046 | 1 | 0.046 | 0.017 | 0.8990 |
| **D-Flow rate (mL)** | 29.18 | 1 | 29.18 | 10.50 | 0.0059 |
| **AB** | 7.01 | 1 | 7.01 | 2.52 | 0.1346 |
| **AC** | 2.97 | 1 | 2.97 | 1.07 | 0.3188 |
| **AD** | 0.012 | 1 | 0.012 | 4.352E-003 | 0.9483 |
| **BC** | 7.02 | 1 | 7.02 | 2.53 | 0.1343 |
| **BD** | 0.18 | 1 | 0.18 | 0.066 | 0.8017 |
| **CD** | 0.040 | 1 | 0.040 | 0.014 | 0.9062 |
| **A^2^** | 21.55 | 1 | 21.55 | 7.75 | 0.0146 |
| **B^2^** | 6.71 | 1 | 6.71 | 2.41 | 0.1426 |
| **C^2^** | 0.014 | 1 | 0.014 | 5.177E-003 | 0.9437 |
| **D^2^** | 53.32 | 1 | 53.32 | 19.18 | 0.0006 |
| **Residual** | 38.91 | 14 | 2.78 |  |  |
| **Lack of fit** | 30.09 | 10 | 3.01 | 1.36 | 0.4100 |
| **Pure error** | 8.82 | 4 | 2.21 |  |  |
| **Total** | 188.63 | 28 |  |  |  |
| **Model summary** | | | | | |
| **Standard deviation** | 1.67 | **R^2^ squared** | 0.7937 |  |  |
| **Mean** | 7.97 | **Adjusted R^2^** | 0.5875 |  |  |
| **C.V (%)** | 20.92 | **Adeq Precision** | 7.484 |  |  |
| Loading capacity=-310.12+4.51A+18.81B+12.60C+150.22D+0.29A*B-0.19A*C+0.12A*D-0.58B*C-0.94B*D-0.44C*D-0.20A^2^-0.45B^2^+0.02C^2^-127.42D^2^ | | | | | |
| *p<0.05 significant, **p<0.01 significant, ns not significant | | | | | |
